# The use of water-soluble phthalocyanines as textile dyes in nylon/elastane fabric: fastness and antibacterial effectiveness

**DOI:** 10.3906/kim-1912-11

**Published:** 2020-08-18

**Authors:** Armağan GÜNSEL, Ayşe USLUOĞLU, Ahmet Turgut BİLGİÇLİ, TOSUN Büşra, Gülnur ARABACI, Meryem Nilüfer YARAŞIR

**Affiliations:** 1 Department of Chemistry, Faculty of Arts and Sciences, Sakarya University, Sakarya Turkey; 2 Aydın Örme Industry and Trade Inc., Sakarya Turkey

**Keywords:** Phthalocyanine, water soluble, naylon/elastane, dye, fastness, antibacterial

## Abstract

This work reports on dyeing of nylon/elastane fabric with water-soluble phthalocyanines ( **1-4** ) bearing quinoline 5-sulfonic acid substituents on the peripheral or nonperipheral positions and determining the antibacterial efficiency of the phthalocyanine compounds and the dyed nylon/elastane fabrics. The light, washing, water, perspiration, and rubbing fastness properties of nylon/elastane fabrics dyed with phthalocyanines were also determined. The results showed that all dyed fabrics showed very good wet fastness values. The lightfastness value of the nylon/elastane fabric dyed with phthalocyanine dye ( **1** ) showed a much better value than the others. Also, the antibacterial efficiencies of the dyed nylon fabrics and the dye compounds were investigated against a gram-negative ( *Escherichia coli* ) and a grampositive ( *Staphylococcus aureus* ) bacteria by using disc diffusion method. The results showed that the dyed nylon/elastane fabrics and the compounds exhibited antibacterial activities against both bacteria.

## 1. Introduction

Dyes are molecules that absorb and reflect light at specific wavelengths to give human eyes the sense of color [1]. There are two major types of dyes-natural and synthetic dyes. The natural dyes are extracted from natural substances such as plants, animals, or minerals. Synthetic dyes are made in a laboratory using chemicals. Some of the synthetic dyes contain metals too. One of the most used applications of dyes is to be used for coloring the fabrics [2]. Textile industry needs many synthetic dyes, such as sulfur dyes, basic dyes, acid dyes, disperse dyes, reactive dyes, direct dyes, and vat dyes, to meet the ever-growing demand for quality, fastness, variety, and color depth [3]. Acid dyes are very common and the most important group of dyestuffs. The presence of sulfonic acid groups (–SO_3_) or nitro (–NO_2_) groups in the molecule gives acidity property to most acid dyestuffs [4]. Water-soluble anionic dyes known as acid dyes are used to dye nitrogenous fibers that have basic groups such as polyamide and wool [5,6].

A dye, whether it is from a natural or synthetic origin, is used, not only to just color the surface of fibers, but it must also become a part of the fiber. After dyeing, the fabric should not be affected during the washing process, dry cleaning with organic solvents, etc. and also the dye should give fastness to light, heat, and bleaching. Textile fibers may be classified as natural, semi-synthetic, and synthetic. The most important completely synthetic fibers are polyester, polyamides (nylon), and acrylic fibers [7]. Nowadays, polyamide fiber is a widely used synthetic fiber. It is generally used in socks, knitted stretch outwear, underwear, swimwear, sportswear, activewear, lace because of its excellent abrasion resistance, strength, and recycling characteristics [8,9]. The use of synthetic fabrics in direct contact with the human skin may be subject to attack by microorganisms and may be a serious problem for bacterial contamination. The moisture and natural properties of these fabrics may be most appropriate for the survival of the microorganisms. Pathogenic microorganisms that are dangerous to human health, especially *Escherichia coli* and *Staphylococcus aureus* , must be prevented from contaminating textile fabrics. For this purpose, it is important to develop new antibacterial textile materials and dyes [10].

The phthalocyanine dyes are a family of dyes that are obtained by the reaction of dicyanobenzene in the presence of metal atom such as copper, nickel, cobalt, etc. The phthalocyanine nucleus gives the molecule a good fastness to light. The most widely used dye in this family is copper phthalocyanine because of its high chemical stability [11–13]. The main limiting factor for phthalocyanines to be used in many areas is their poor solubility in solvents. A phthalocyanine must have a solubility in the application medium, generally water, at some points during the coloration process for the textile industry [14,15]. The solubility can be modified either by inserting different metal atoms into the inner core of the Pc ring or by substitution of functional groups at the peripheral/nonperipheral regions of the Pc ring with anionic or cationic groups, known as auxochromes (color helpers), examples of which are sulfonic acid, carboxylic acid, or quaternized ammonium groups. While these functional groups are not responsible for color, at the same time, are used to shift the color of a colorant and/or affect the dye solubility [16,17].

Phthalocyanine compounds are capable of dyeing textile fabrics which exhibit significant biological activities such as antimicrobial and antioxidant activities based on their specific structures. However, there is not much work regarding the antibacterial activity of the dyed nylon fabric with phthalocyanine compounds. Several researchers investigated the physical, mechanical, and antibacterial properties of the textile fabric treated with some antibacterial dyes and stuffs [18–20].

In the present study, the nylon/elastane fabric was dyed with water-soluble phthalocyanines ( **1-4** ) bearing quinoline 5-sulfonic acid substituents on the peripheral or nonperipheral positions and the fastness and dyeing properties of the dyed fabric were examined. Additionally, the antibacterial efficiencies of the compounds andthe dyed nylon/elastane fabrics were evaluated for the first time in this study.

## 2. Experiment

### 2.1. Materials and methods

1(4), 8(11), 15(18), 22(25)-Tetrakis(8-(2,3-dicyanophenoxy) quinoline-5-sulfonic acid)-copper (II) phthalocyanine ( **1** ), 1(4), 8(11), 15(18), 22(25)-Tetrakis(8-(2,3-dicyanophenoxy) quinoline-5-sulfonic acid)-cobalt (II) phthalocyanine ( **2** ), 2(3), 9(10), 16(17), 23(24)-Tetrakis(8-(3,4-dicyanophenoxy) quinoline-5sulfonic acid)-copper (II) phthalocyanine ( **3** ) and 2(3), 9(10), 16(17), 23(24)-Tetrakis(8-(3,4-dicyanophenoxy) quinoline-5sulfonic acid)-cobalt (II) phthalocyanine ( **4** ) were prepared according to the procedure in literature (Schemes S1 and S2) [21]. 80% Nylon 6- 20% Elastane knitting fabric, 100g/m^2^ , ready fabric for dyeing was supplied Aydın Örme Industry and Trade Inc. (İstanbul, Turkey), leveling agent - Laugal TP (Erca Group Chemistry Industry and Trade Inc., Tekirdağ, Turkey), acetic acid, laboratory dyeing machine – Ecodyer, GretagMacbeth Colour- Eye 7000A spectrophotometer, incubator, grayscale, crockmeter, multifiber, ECE phosphate test detergent B, l-histidine monohydrochloride monohydrate, Sodium chloride, Disodium hydrogen orthophosphate dihydrate, sodium hydroxide.

### 2.2. The dyeing process

**Figure 1:**
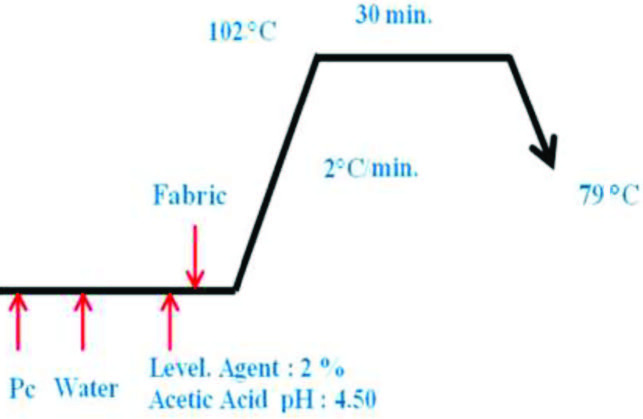
Dyeing process of nylon/elastane fabric (NEF).

A dyeing bath of 1/20 ratio and 5 g of nylon/elastane fabric was used. Phthalocyanines were applied at 1% concentration to the fabric. 0.05 g of phthalocyanine (1-4), 0.6 g/L of the acetic acid solution, leveling agent (Laugal TP) solution 2%, and 97.4 mL of water was added to a dyeing tube. The dye bath is maintained at a pH of 4.50. The temperature was raised to 102 ° C over 30 min and held at this level for 30 min. Then, the dyed fabrics were removed, rinsed with water, and dried at 60 ° C for 10 min in an oven (Figure 1). The dyeing process of the fabric with all dyestuffs was performed three times.

### 2.3. Color measurements

CIE L*a*b* (CIELAB) is a color space indicated by the International Commission on Illumination (French Commission internationale de l’éclairage, hence its CIE initialism). It defines all the colors visible to the human eye and was produced to serve as a device-independent model to be used as a reference [22]. The measurement was performed by the spectrophotometer (GretagMacbeth Colour-Eye 7000A) with the parameter of D65 daylight and 10◦ standard observer. Color space defined by the CIE, based on 1 channel for Luminance (lightness) (L) and 2 color channels (a and b).The CIE L*a*b* values were taken and the results of the color properties of dyestuffs under the dyeing condition. The L* axis represents the lightness of a sample. L* = 0 (black), L* = 100 (white); a* represents the redness or greenness of a sample +a*value = red, - a* value = green. The higher a* value means redder and lower a* value means greener; b* represents the blueness or yellowness of a sample +b* value = yellow, - b* value = blue. The higher b*value means yellower and lower b* value means bluer. The c* axis represents Chroma or ’saturation’. The h* axis represents Hue. The tests were done in triplicate and mean values are given in Table 1.

**Table 1:** CIELAB values for dyeing of nylon/elastane fabric (NEF) with phthalocyanine. (L* = Luminance (lightness), a* = Redness or greenness of a sample, b* = Blueness or yellowness of a sample, c* = Chroma or ’saturation’, h* = Hue). NEF1 = Dyed with the compound 1, NEF2 = Dyed with the compound 2, NEF3 = Dyed with the compound 3, NEF4 = Dyed with the compound 4.

		L*	a*	b*	c*	h°	K/S
(NEF1)	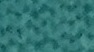	64.34 ± 0.28	–29.08 ± 0.2	–3.08 ± 0.11	29.24 ± 0.25	186.05 ± 0.15	10.283 ± 0.14
(NEF2)	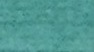	75.31 ± 0.32	–25.75 ± 0.2	–0.66 ± 0.12	25.76 ± 0.2	181.47 ± 0.15	2.82 ± 0.12
(NEF3)	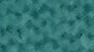	66.63 ± 0.21	–27.90 ± 0.29	–1.20 ± 0.14	27.93 ± 0.27	182.46 ± 0.11	6.38 ± 0.16
(NEF4)	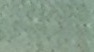	78.25 ± 0.32	–14.21 ± 0.26	4.94 ± 0.19	15.05 ± 0.151	160.84 ± 0.16	1.254 ± 0.11

### 2.4. Fastnesses testing

The dyed nylon/elastane fabrics were evaluated according to ISO standard methods: ISO 105-X12:2002 for colorfastness to rubbing; ISO 105-C06:2012 for colorfastness to washing and colorfastness, BS EN ISO105 E01:2013 for water fastness, ISO 105 E04: 2013 for Perspiration, BS EN ISO 105 - B02:2014) 1999 for lightfastness. For washing, rubbing, water, perspiration fastness measurements, staining, and changes in shade were assessed with the grayscale [ISO 105 A03]. The fastness tests were performed in triplicate and are presented in Table 2.

**Table 2:** Fastness Properties for dyeing of nylon/elastane fabric (NEF) with phthalocyanine.

	Light	Washing	Water	Perspiration		Rubbing	
				Alkali	Acid	Dry	Wet
(NEF1)	5	5	5	5	5	5	5
(NEF2)	4	5	5	5	5	5	5
(NEF3)	4	5	5	5	5	5	5
(NEF4)	3/4	5	5	5	5	5	5

### 2.5. Antibacterial affectivity tests

The disc diffusion method had been applied to the phthalocyanine compounds, the dyed nylon/elastane fabrics and nylon/elastane fabric for the antibacterial tests. For this purpose, a gram-negative [ *Escherichia coli* (ATCC 25922)] and a gram-positive ( *Staphylococcus aureus* (ATCC 29213)) bacteria were used. The disk diffusion assay was performed as previously described [23, 24, 25].

**Figure 2:**
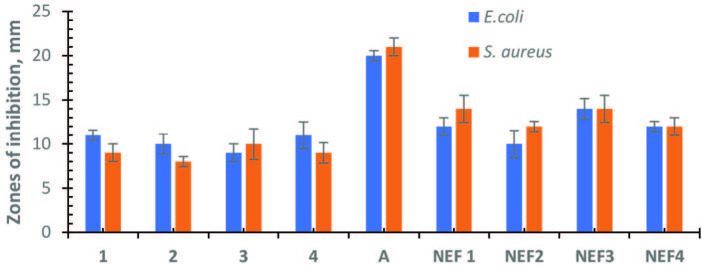
Antibacterial effectiveness of all tested material and Ampicillin (A) standard against *Escherichia coli* and *Staphylococcus aureus* bacteria strains. *Inhibition zone diameter in millimeters. NEF1 = Dyed with the compound **1** , NEF2 = Dyed with the compound **2** , NEF3 = Dyed with the compound **3** , NEF4 = Dyed with the compound **4** .

In disc diffusion method, Mueller-Hinton agar plates were prepared, sterilized, and then inoculated with bacterial strains. 20 μL solutions of the compounds in water at a concentration of 500 mg/L were added to the sterile blank test discs, then dried and placed on the Mueller-Hinton agar plates. The washed and dried nylon/elastane and the dyed nylon/elastane fabrics were applied directly to the agar plates. The ampicillin disc (10 mg/disc) was used as a standard antibiotic. All prepared agar plates were incubated at 37° C for 24 h. After the incubation period was completed, the inhibition zone diameters were determined in mm. The tests were done in triplicate and mean values are given in Figure 2 (s.d. < 5%).

## 3. Result and discussion

### 3.1. Color measurements of dyed fabrics

The main aim of this work was to show the dyeing ability of the water-soluble phthalocyanine compounds ( **1-4** ) which had quinoline 5-sulfonic acid substituents on the peripheral or nonperipheral positions on nylon fabric (Scheme). The value of CIELAB for dyeing of nylon/elastane fabric with different phthalocyanine compounds were determined to show the quality of dyeing properties of the tested compounds ( **1-4** ). The results were shown in Table 1. According to the results in Table 1, nonperipheral phthalocyanine dyes ( **1** and **2** ) applied to nylon/elastane material presented darker and more vivid colors than dyes of peripheral phthalocyanine dyes ( **3** and **4** ). Phthalocyanine dyes ( **1** and **3** ) had formed darker, more blue and brighter colors than phthalocyanine dyes ( **2** and **4** ). Phthalocyanine dyestuff ( **4** ) formed the lightest (L * 78,25), most matt (c * 15.05), and most yellowish (b * 4.94) colors when compared to other metallophthalocyanine dyestuffs. Copperbased phthalocyanine dyestuff ( **1** ) had the darkest (L * 64.34), brightest (29.24) and most blue (b * –3.08) ranges of the other phthalocyanine dyestuffs. According to the L * values ??in Table 1, the colors of the fabrics dyed with the phthalocyanine dyes containing copper metal atom were observed to be darker and bright green. Also, the nonperipheral substitution caused the fabrics dyed with both copper and cobalt metallophthalocyanine dyes to be a darker color than the others. The values of a *, b *, c * in Table 1 showed that the fabric dyed with cobalt metallophthalocyanine dyes containing substituents at the peripheral positions had much lighter and less green color than all dyed fabrics. According to K/S values in Table 1, the fabrics dyed with copper metallophthalocyanines were observed to be darker compared to those dyed with cobalt metallophthalocyanines. The h◦ values of the compounds ( **1-3** ) were found to be 186.05, 181.47, and 182.46, respectively. But, the h◦ value of the compound ( **4** ) was observed to be 160.84. Therefore, the compounds ( **1-3** ) gave the fabric a bluish-green color, while the compound ( **4** ) gave the fabric a yellowish-green color (Figure S1).

**Scheme:**
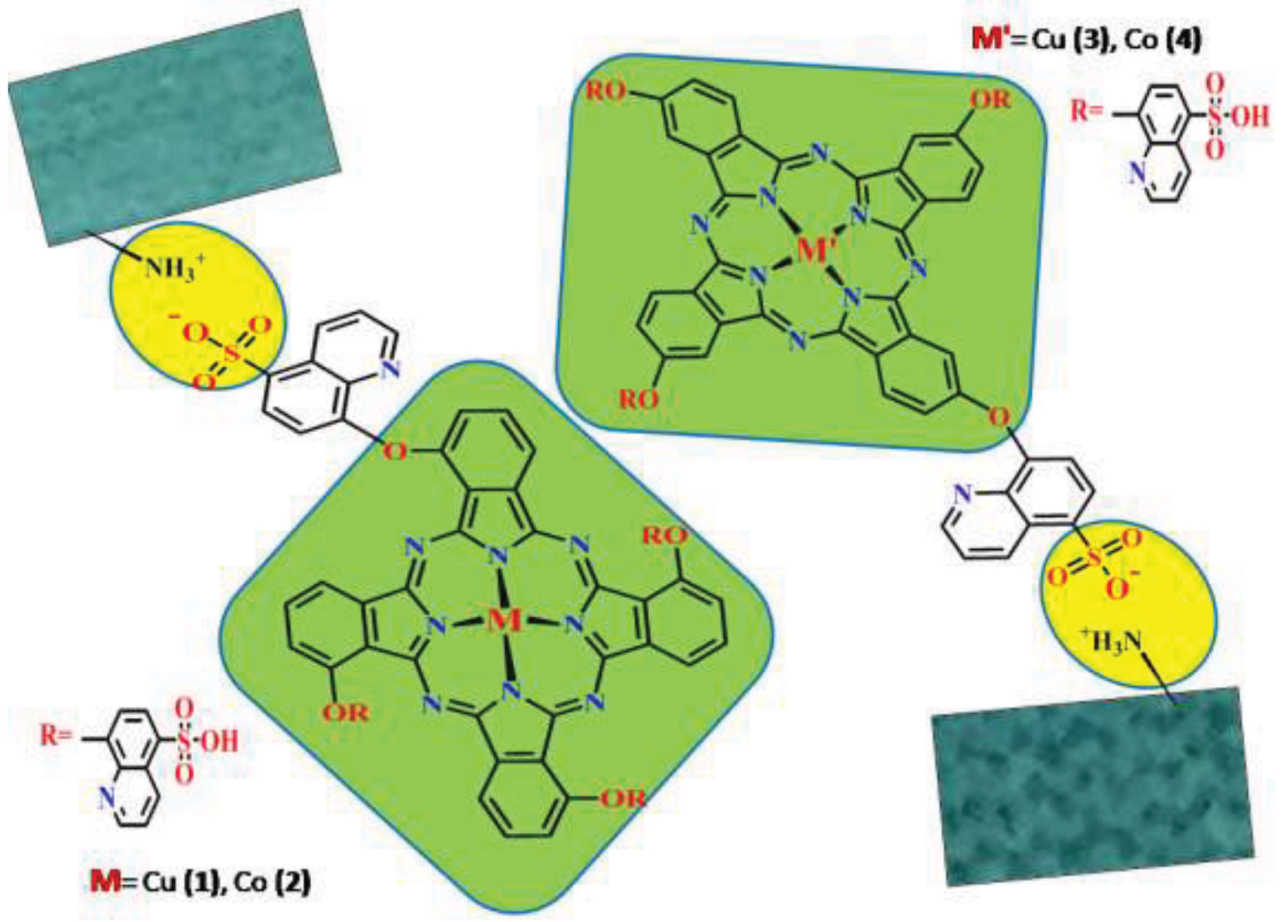
Structure of water-soluble phthalocyanines ( **1-4** ) bearing quinoline 5-sulfonic acid substituents on the peripheral or nonperipheral positions.

### 3.2. Fastness properties

Colorfastness is the most important feature that must be set to show the quality of the dyed materials. Fastness is defined as the strength of the color against many factors during the production and use of the textile material [26].

In this study, the light, washing, water, perspiration, and rubbing fastness values of nylon/elastane fabrics dyed with four different phthalocyanine dyes ( **1-4** ) were determined to show the dyeing quality of the phthalocyanine dyes. The results were demonstrated in Table 2. All dyed fabrics showed very good wet fastness properties as demonstrated in Table 2. There were no visible staining and color change on the multifiber and the dyed nylon/elastane specimen. The lightfastness of dyed nylon/elastane fabric with phthalocyanine dyestuff ( **1** ) had the best value with 5 when it was compared to the other dyed fabrics. The results of phthalocyanine dyestuffs ( **2** and **3** ) are the same, with 4 values. The lightfastness value of the phthalocyanine dyestuff ( **4** ) was determined as 3/4.

### 3.3. Antibacterial effectivity assessment

In this study, the antibacterial efficiencies of the water-soluble phthalocyanine compounds and the dyed nylon/elastane fabrics were evaluated with the disc diffusion method. The antibacterial effectiveness of the Pc compounds in the dyed and undyed fabrics was determined against Gram-negative ( *E. coli* ) and Gram-positive ( *S. aureus* ) bacteria (Figures S2–S4). Ampicillin was used as a positive standard for both bacterial strains (Figure S5). The inhibition zones (mm) for each sample were measured and presented in Figure 2. The phthalocyanine dye compounds and the nylon/elastane fabric dyed with these phthalocyanine compounds showed an antibacterial effect against the bacteria tested. However, the undyed nylon/elastane fabric used as a control did not show any antibacterial activity to the bacteria. All phthalocyanine compounds showed reasonable antibacterial activity against *E. coli* and *S. aureus* with 8-11 mm inhibition zones. Compound 3 had slightly better antibacterial activity than the other phthalocyanine compounds. Also, the dyed nylon/elastin fabrics were also evaluated for their antibacterial effectiveness. According to the results in Figure 2, the fabric dyed with the compound ( **3** ) had slightly better antibacterial activity against both bacteria than those dyed with compounds ( **1** ), ( **2** ), and ( **4** ). It can be concluded that the nylon/elastane fabric dyed with phthalocyanine compounds shows reasonable antibacterial activity as we expected. Because the synthesized phthalocyanine compounds also carried antibacterial activity as seen in Figure 2. There are several studies in the literature about metal-coated nylon fabrics such as silver [24] and copper [27] with UV protection, electroconductivity, and antibacterial properties. Our results showed parallel results with the literature on nylon fabric with antibacterial activity.

## 4. Conclusion

In the present study, the nylon/elastane fabric was successfully stained with four different water-soluble phthalocyanine ( **1-4** ) compounds for the first time in the literature. The property of fastness was determined to demonstrate their dyeing qualities. The wet fastness qualities of all tested phthalocyanine dyestuffs were very good. The lightfastness value of the phthalocyanine dye ( **1** ) has also a very good value compared to the others. Also, the antibacterial effectiveness of the water-soluble phthalocyanine compounds and the dyed nylon/elastane fabric were evaluated against *E. coli* and *S. aureus* bacteria for the first time. They all showed significant antibacterial properties. The positive results of this study reveal that the new phthalocyanine dyestuffs that can dye nylon fibers in green tones with good light, wet fastness, and antibacterial effect have been obtained.

Supplementary Materials
