# Supplementary material for: The use of water-soluble phthalocyanines as textile dyes in nylon/elastane fabric: fastness and antibacterial effectiveness
Source: Turk J Chem. 2020 Aug 18;44(4):923–31. doi: 10.3906/kim-1912-11 (PMC7751918; doi:10.3906/kim-1912-11)
Supplement: Supplementary file 1 — Supplementary Materials [file turkjchem-44-923-sup001.pdf]

Supplementary Material

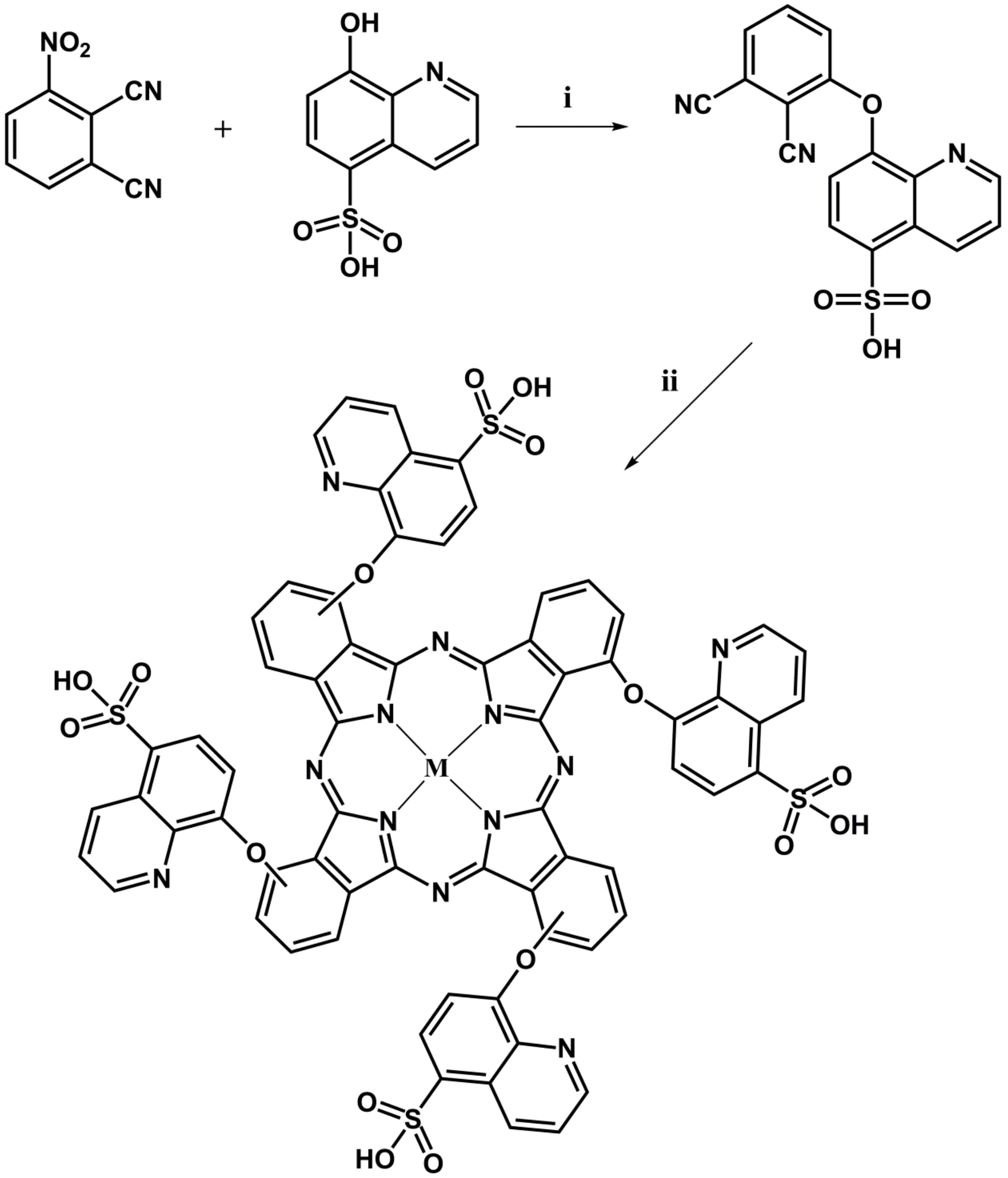

M: Cu **(1)**; Co **(2)**

Scheme S1. Synthetic route of 1(4), 8(11), 15(18), 22(25)-tetrakis 8-hydroxyquinoline-5-sulfonic acid (HQSA) phthalocyanines (M: Cu (II) **(1)**, Co(II) **(2)**). **i.** K_2CO_3, DMF, 45 °C, **ii.** Metal salt ($CuCl_2$, $CoCl_2$), N, N-dimethyl amino ethanol (NNDMAE), 1.8-diazabicyclo [5.4.0] undec-7-ene (DBU), 10h.

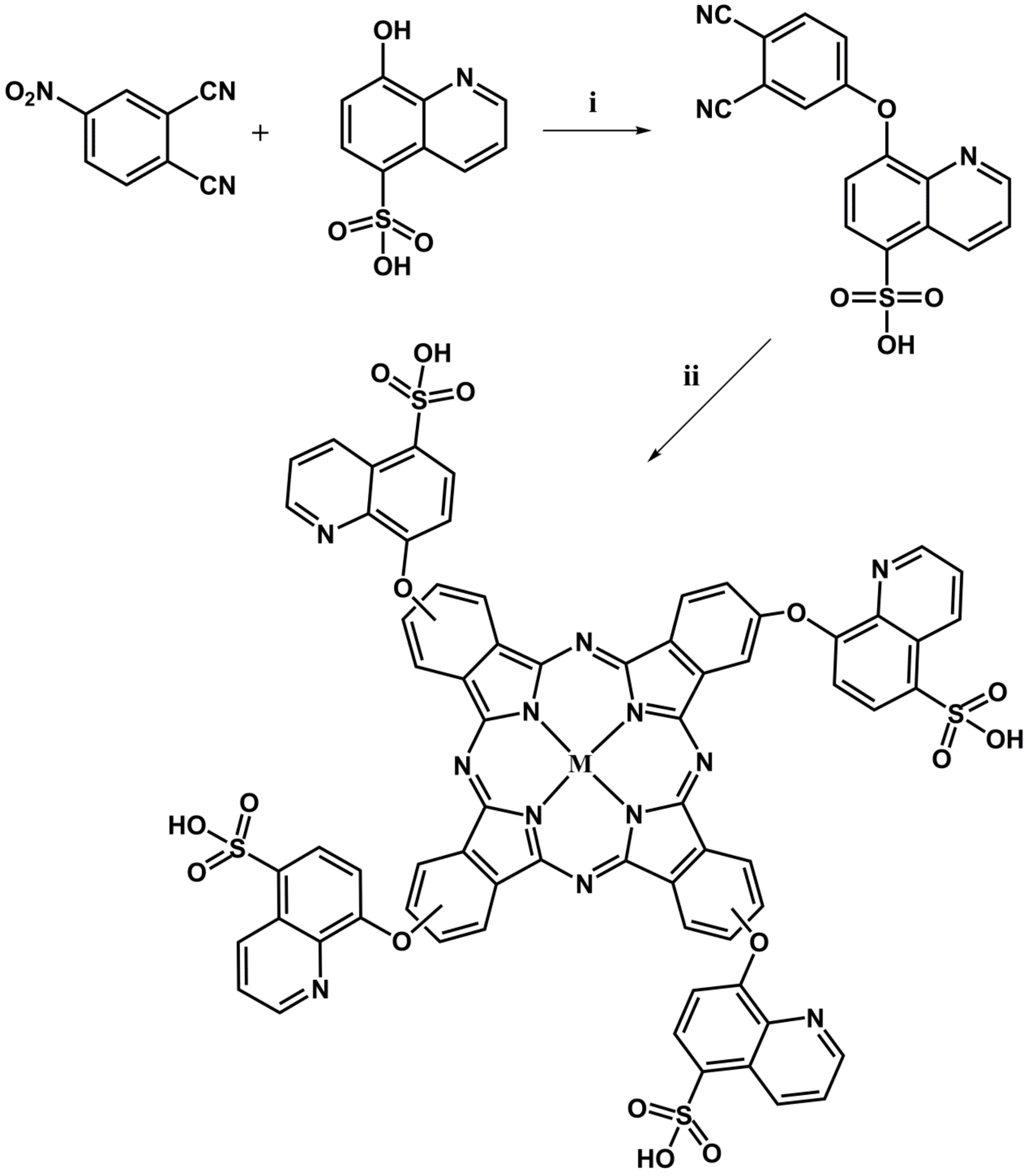

M: Cu **(3)**; Co **(4)**

Scheme S2. Synthetic route of 2(3),9(10),16(17),23(24)-tetrakis 8-hydroxyquinoline-5-sulfonic acid phthalocyanines (M: Cu (II) **(3)**, Co (II) **(4)**), **i.** K_2CO_3, DMF, 45 °C, **ii.** Metal salt ($CuCl_2$, $CoCl_2$), N, N-dimethyl amino ethanol (NNDMAE), 1.8-diazabicyclo [5.4.0] undec-7-ene (DBU), 10h.

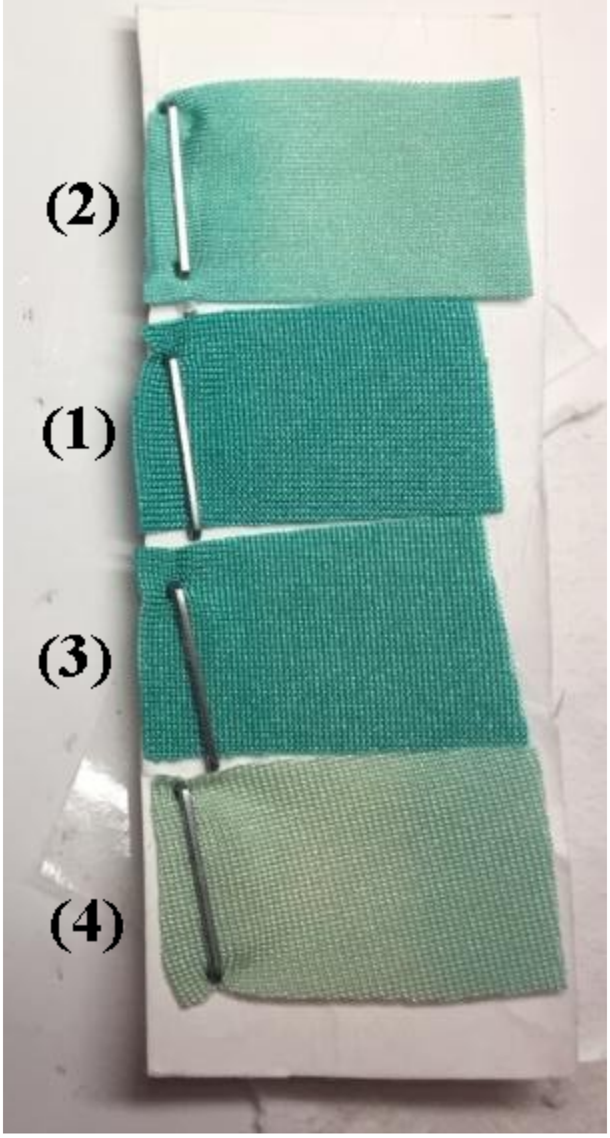

Figure S1. The original photos of the nylon/elastan fabrics dyed with the metallophthalocyanines **(1-4)**

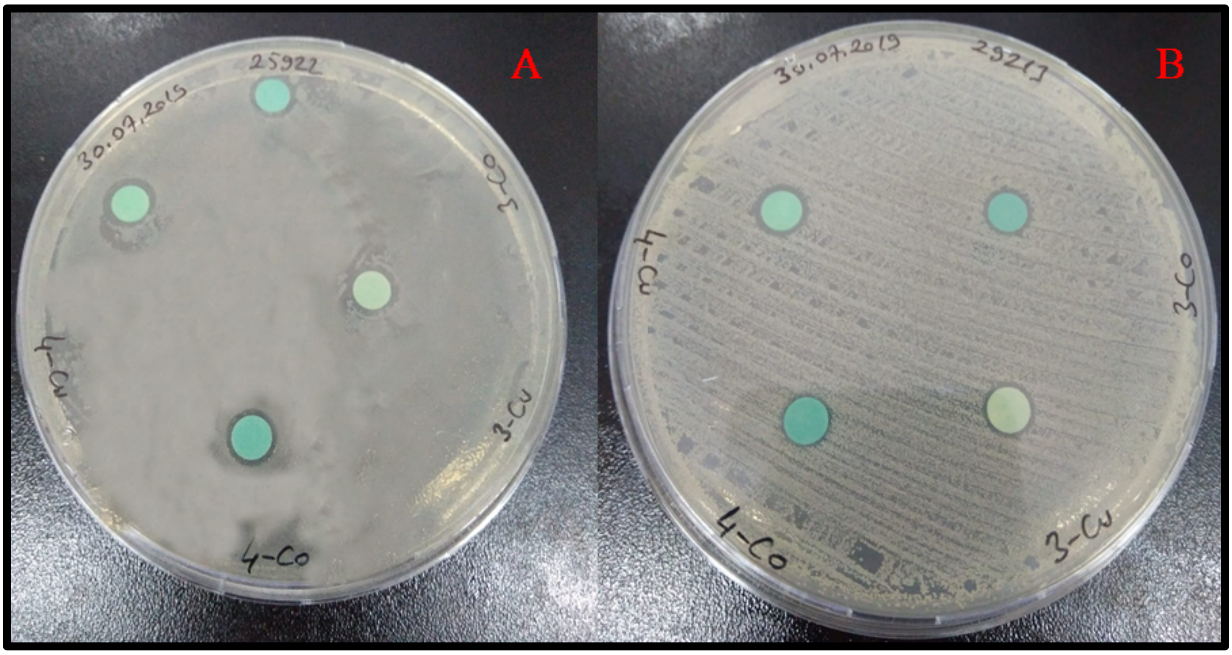

Figure S2. Antibacterial activities of phthalocyanines compounds **1** (3Cu), **2** (3Co), **3** (4Cu) and **4** (4Co) against *Escherichia coli* (ATCC 25922) (A) and *Staphylococcus aureus* (ATCC 29213) (B) with disc diffusion method.

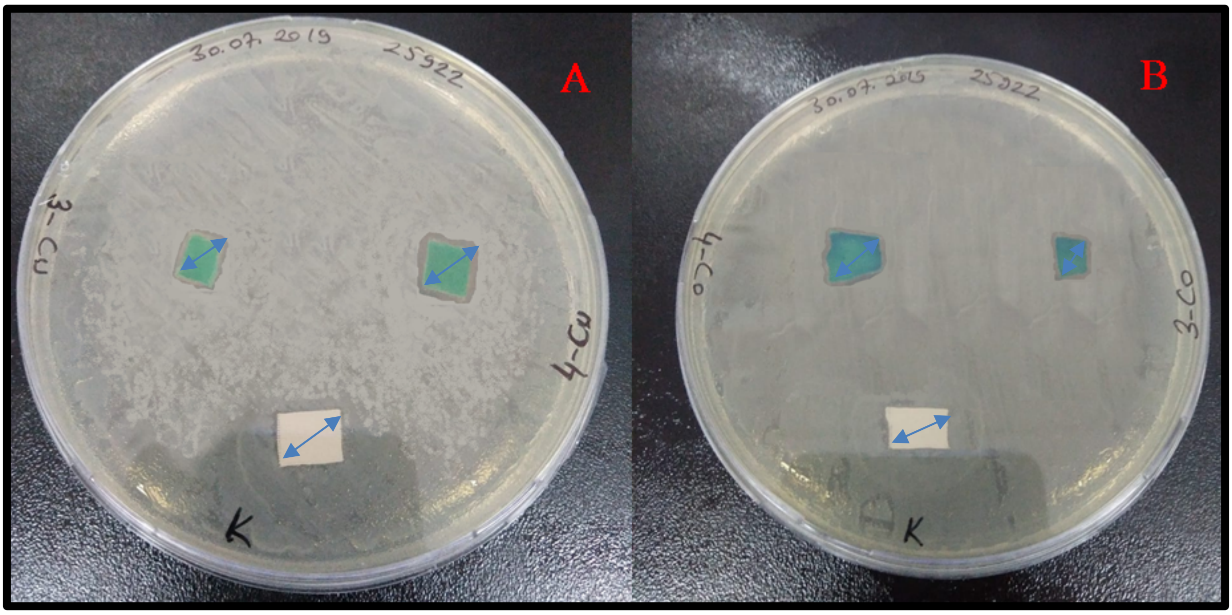

Figure S3. Antibacterial activities of the dyed nylon/elastan fabric (NEF) with phthalocyanine compounds **1** (3Cu), **3** (4Cu) (A) and **2** (3Co), **4** (4Co) against *Escherichia coli (ATCC 25922)*.

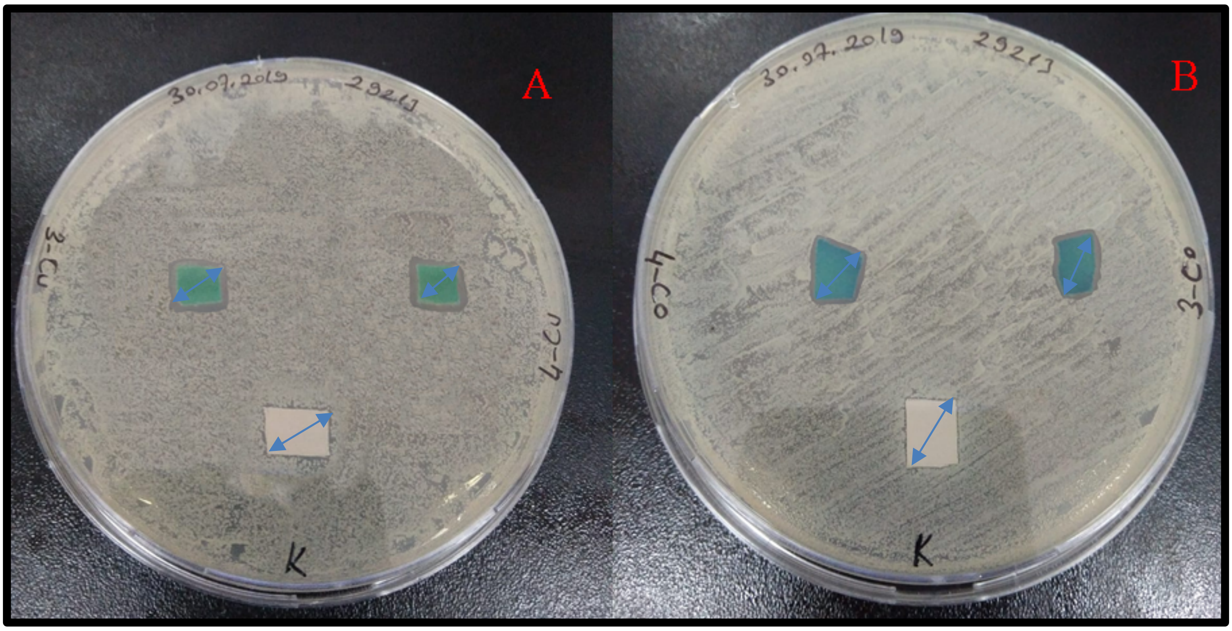

Figure S4. Antibacterial activities of the dyed nylon/elastan fabric (NEF) with phthalocyanine compounds **1** (3Cu), **3** (4Cu) (A) and **2** (3Co), **4** (4Co) against *Staphylococcus aureus* (ATCC 29213).

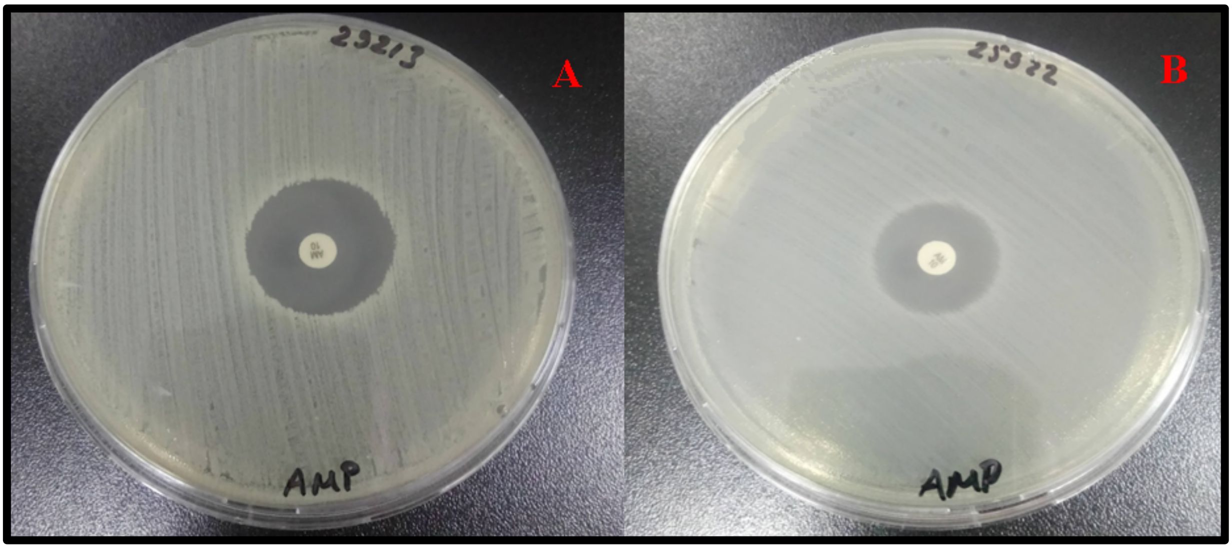

Figure S5. Antibacterial results of standard antibitotic Ampicillin (AMP) against *Staphylococcus aureus (ATCC 29213)* (A) and *Escherichia coli (ATCC 25922)* (B) with disc diffussion method.
